# Transfusion Rates in Free Flap Breast Reconstruction Patients: A Single-Centre Experience

**DOI:** 10.7759/cureus.76796

**Published:** 2025-01-02

**Authors:** Oliver Blanshard, Lucinda Knight, Toby Noton, Fateha Chowdhury, Georgina Williams

**Affiliations:** 1 Plastic and Reconstructive Surgery, Imperial College Healthcare NHS Trust, London, GBR; 2 Hematology, Imperial College Healthcare NHS Trust, London, GBR

**Keywords:** autologous breast reconstruction, breast reconstruction surgery, deep inferior epigastric perforator flap, group and save, post-operative haemorrhage, pre-operative assessment, transfusion requirements, transverse upper gracillis flap

## Abstract

Background

Previous studies have demonstrated low transfusion rates in breast reconstruction with deep inferior epigastric perforator (DIEP) flaps. We often employ the transverse upper gracilis (TUG) flap; however, perioperative transfusion rates have not previously been studied in this group. Due to the different dissection and exposure required, transfusion rates may vary.

We aim to ensure that perioperative resource use is appropriate and efficient, particularly group and save (G&S) sampling preoperatively. The objective of this study is to quantify transfusion rates in all patients undergoing free flap-based breast reconstruction and to determine the necessity of preoperative G&S sampling.

Methods

We retrospectively reviewed the electronic patient records, the electronic transfusion system, operation notes, and prescription records of all patients undergoing breast reconstruction using free-flap tissue transfer over one year. We identified transfusion of red blood cells and the current practice of perioperative G&S sampling in this patient group. These data were analysed using descriptive statistics.

Results

Of the 124 patients undergoing breast reconstruction with a free flap, a DIEP-based flap was deployed in 105, and a TUG flap in 14; in the remaining five, a selection of other flaps were utilised.

Three patients required transfusion of blood products during their admission (2.4%), all after DIEP flap-based reconstructions. All received two units of packed red blood cells, with one transfusion on each of days one to three postoperatively. The indication for all three was slow but ongoing bleeding with low haemoglobin (less than 80 g/L) on routine full blood count. All recovered well following this.

We found a low rate of transfusion in patients undergoing free flap breast reconstruction, with only three of 124 requiring transfusion (2.4%), all DIEP flaps. In addition to the published literature regarding DIEP flaps, we also include several TUG flaps in this cohort and several other flap types. None of the three transfusions were emergent in nature; all were completed with fully cross-matched blood according to local protocols.

Conclusion

We recommend that preoperative G&S sampling is not routinely necessary for patients undergoing free flap breast reconstruction. Preoperative G&S should be considered for those with a risk of atypical anti-red cell antibodies (for example, if previously transfused or pregnant), as cross-matched blood may take several days to be made available.

We expect this judicious use of G&S sampling to significantly reduce costs and laboratory resource use without a significant effect on the use of emergency red cell units.

## Introduction

Following oncological mastectomy, breast reconstruction is commonly performed. Options for breast reconstruction most commonly include insertion of a breast implant or free-flap reconstruction as the second most common approach [1]. Free flap-based reconstruction is recognised to result in better aesthetic outcomes, with patients perceiving the reconstructed breast as more natural [2]. It is, however, more costly [2]. At our centre in London, United Kingdom, free flap-based breast reconstruction is performed by the Plastic and Reconstructive Surgery team in collaboration with the Breast Surgery team, who perform the oncological resection. Free flap-based reconstruction can be performed immediately, in the same sitting as the oncological resection, or as a delayed operation. Within free flap-based breast reconstruction, the deep inferior epigastric perforator (DIEP) flap is most frequently employed. At our centre, the transverse upper gracilis (TUG) flap is used in approximately one in 10 free flap reconstructions, and the transverse rectus abdominis muscle (TRAM) flap in fewer cases.

Free flap-based breast reconstruction is considered "major surgery" [3]. Thorough preoperative assessment is crucial to evaluate modifiable and non-modifiable perioperative risk factors for patients and to gather adequate information on individual patients for operative planning. This includes clinical assessment of the donor site for choice of flap, computed tomography (CT) evaluation of the deep inferior epigastric artery and its perforators, and routine blood testing for full blood count and renal function [3]. Risk stratification and preparation for potential blood loss and red blood cell transfusion also occur at this stage.

This study focuses on group and save (G&S) screening. G&S screening involves typing the patient’s blood within the ABO and RhD groups and screening the serum for atypical anti-red cell antibodies (AARCAbs), which are antibodies against allogenic red cell antigens, excluding the common anti-ABO and anti-RhD antibodies. Two G&S samples are used by the transfusion laboratory to crossmatch red blood cell units in the event of red cell transfusion. In the absence of any red cell antibodies, electronic issue is acceptable (i.e., release of ABO- and Rh-compatible red cells without the need for formal crossmatch). Crossmatched blood must be used for transfusion if antibodies are present, both to prevent transfusion reactions and to reduce the risk of delayed haemolytic transfusion reactions and the development of further antibodies. Where possible, avoiding the use of emergency blood stocks reduces reliance on these scarce resources [4].

The recommendation for preoperative G&S screening for each operation varies across the UK and is based on both guidelines and local experience (local guidelines are available online from individual Trusts as "maximum surgical blood order schedules") [5,6]. Our local guideline currently recommends arranging four units of crossmatched RBCs preoperatively for mastectomy and immediate free flap reconstruction; actual practice is suspected to vary and has not been recently assessed. To reduce the need for transfusion, and in line with national guidelines, tranexamic acid is routinely used [7]. Cell salvage is rarely used in these cases as it is contraindicated at the cancer site, limiting its use to the site of flap harvest [8].

In this study, we aimed to quantify the transfusion rate in patients undergoing free flap breast reconstruction and assess the rate of preoperative G&S sampling. We include a number of TUG flaps in this study, in contrast to previously published literature that reports only on DIEP flaps. We explore each case that required transfusion and describe our conclusions regarding the use of preoperative G&S sampling.

## Materials and methods

Patient identification and screening

This was a retrospective review of transfusion rates at a secondary breast reconstruction centre over the course of one year, from January 1, 2023, to December 31, 2023. Patients were identified from the electronic theatre records, inpatient lists, and scheduling lists. These records contained a complete list of all operations completed within the hospital network and were cross-referenced to ensure no patients were missed. Patient records were subsequently screened against the inclusion and exclusion criteria. All patients who underwent immediate or delayed breast reconstruction with a free flap during the study period of one year were included. Breast reconstructions performed at this secondary referral centre included DIEP flaps, TUG flaps, TRAM flaps, and superficial inferior epigastric artery (SIEA) flaps. We excluded patients undergoing breast reconstruction with a non-free flap, namely latissimus dorsi flap reconstructions. There were no incomplete records or incomplete data.

Data collection

Operative details were collected from the electronic operation report and operative documentation on the electronic patient record. These included laterality, flap type, type of reconstruction, and any contemporaneous resection (mastectomy with or without lymph node biopsy or clearance). Lymph node status was also recorded. Transfusion requirements were ascertained from electronic laboratory reports and prescription records on the electronic patient records. Preoperative medical factors, including oncological treatment given, history of cardiac, respiratory, and haematological disorders, and diagnosis of diabetes, were obtained from the electronic patient records. All transfusions during the index admission were included, and all transfusions were given in whole-unit increments. Patients’ preoperative blood test results were collected from the electronic patient records.

Data analysis

Demographic and baseline data were analysed within the data collection tool, Microsoft Excel (Excel for Windows 2016, Microsoft Corporation, Redmond), using descriptive statistics, including mean, median, standard deviation, and percentages. Demographics analysed included patients’ dates of birth, gender, and the American Society of Anaesthesiologists (ASA) perioperative morbidity grade [9]. Group and save sampling rates, operative details, and any red blood cell transfusions given were also recorded and analysed directly within the database.

Data storage and anonymisation

Once patients were identified, screened, and data collected, the database was anonymised by removing all identifiable information, including hospital identification numbers, dates of birth, and all other personal identifiers. All study data were stored on Trust PCs, with a backup on Trust-provided storage, according to local protocols. Data were transferred, where absolutely necessary, via the Trust-provided secure data transfer portal. Data were processed in accordance with relevant legislation, namely The Data Protection Act 2018, as well as national and local regulations.

Ethics approval

All data recorded in the patient records were part of routine clinical care, documented at the time of each patient’s inpatient hospital admission. Local approval for data collection and analysis for this project was granted via the Trust’s Audit and Service Evaluation Team; due to its retrospective nature, formal ethics approval was not required.

## Results

Operative and demographic data

**Figure 1:**
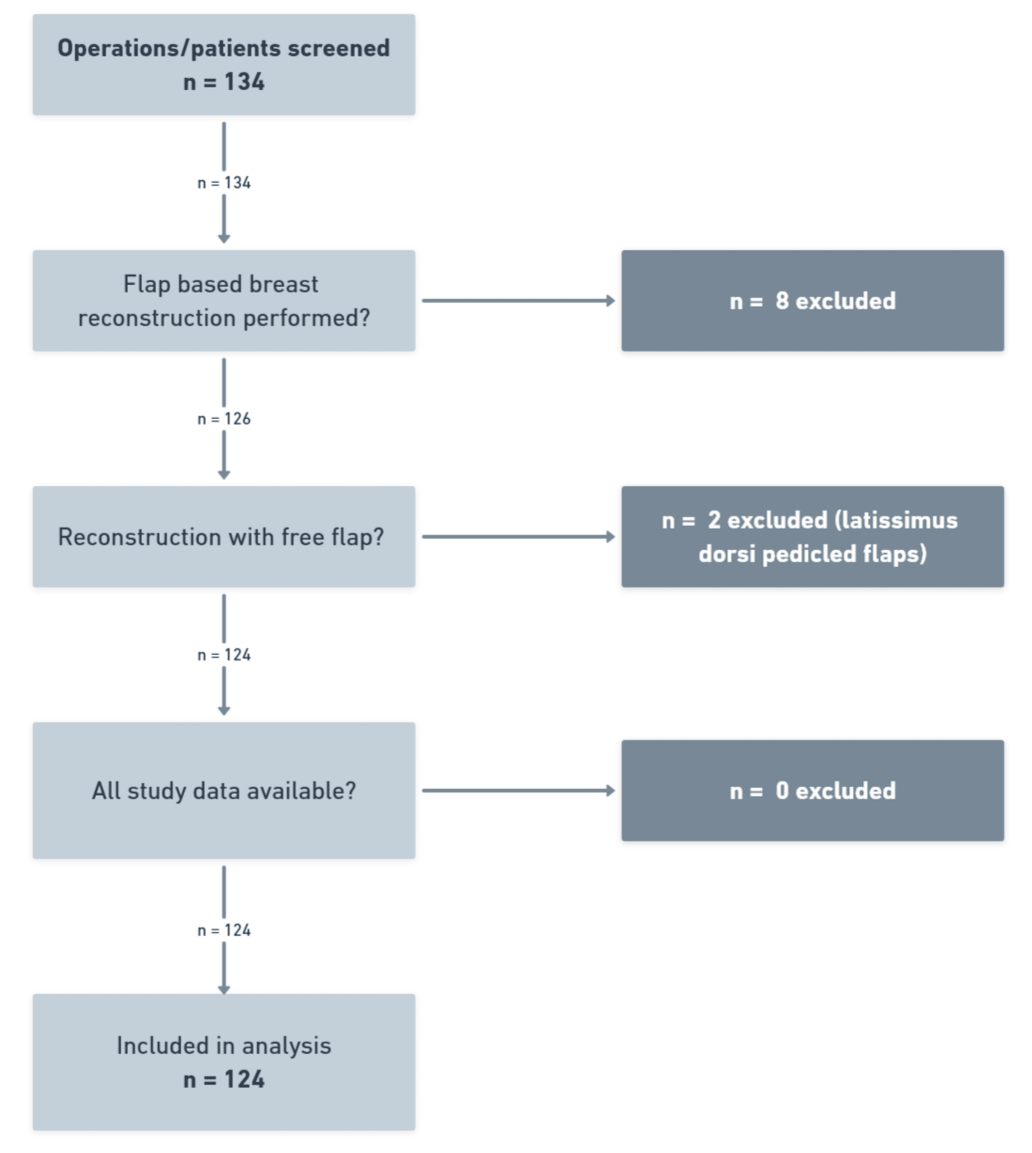
Flow diagram of study patients

We screened a total of 134 patients listed for breast reconstruction operations, of which 8 were excluded due to not being breast reconstruction operations; two were excluded as they were not free flap-based reconstructions, and all 124 remaining were included in the final analysis. The study flow is demonstrated in Figure 1.

We identified a total of 124 patients who underwent breast reconstruction with a free flap within the study period. All patients were female. Of these, 105 underwent DIEP flaps, 14 had TUG flaps, two had SIEA flaps, one had a transverse rectus abdominis myocutaneous flap, one had a profunda artery perforator flap, and one had an anterolateral thigh-based flap. A full list of operations performed is detailed in Table 1. The average age at the time of operation was 51.1 years (mean, standard deviation = 9.8 years; median 50.0 years). All cases were performed by one of five consultant surgeons, with cases per consultant ranging from 16 to 37. The American Society of Anesthesiologists (ASA) grade was two (“patient with mild systemic disease”) for 121 patients, with two patients having an ASA grade of three (“patient with severe systemic disease”) and one patient having an ASA grade of one (“normal healthy patient”) [9].

**Table 1:** Operative data for all patients DIEP: deep inferior epigastric perforator, TUG: transverse upper gracilis, SIEA: superficial inferior epigastric artery, TRAM: transverse rectus abdominis musculocutaneous, SLNB: sentinel lymph node biopsy.

Flap Type	Total	Laterality (Left)	Laterality (Right)	Bilateral	SLNB	Axillary Clearance
DIEP	105	45	51	9	46	24
TUG	14	6	6	2	7	6
SIEA	2	1	1	0	0	0
TRAM	1	1	0	0	1	0
Profunda artery perforator	1	0	1	0	0	0
Antero-lateral thigh reconstruction of recurrence	1	1	0	0	0	0
Total	124	54	59	11	54	30

Transfusion rates

Of the 124 patients undergoing free flap breast reconstruction, three required red blood cell transfusion (2.4%). All three cases were transfused more than 24 hours postoperatively, each with two units of packed red blood cells. None were emergent transfusions, nor did any require the major haemorrhage protocol or specialist haematology input. These cases are discussed in more detail below. During the respective initial operations, no significant intraoperative complications occurred. No patients experienced flap failure. All received venous thromboembolism prophylaxis with low molecular weight heparin postoperatively from day one onwards, comprising 40 mg of Clexane administered subcutaneously once per day in the evening. All had this held during the 24-hour period in which they received a transfusion. There was no difference in treatment between those requiring transfusion and those not requiring transfusion. None of the three patients who received a transfusion underwent preoperative group and save sampling.

Preoperative medical factors were assessed for all patients receiving transfusion versus those who did not. These are depicted in Table 2. There was no statistically significant difference in any factor between those who did receive a transfusion and those who did not.

**Table 2:** Preoperative factors in those who received post operative transfusion verses those not transfused. No statistically significant difference between groups was identified using the t-test for haemoglobin level and chi-squared test for all other variables. ASA: American Society of Anaesthesiologists.

Pre-operative Medical Factors	Transfused	Not Transfused	Total
Preoperative chemotherapy	1	26	27
Preoperative radiotherapy	0	10	10
Preoperative endocrine therapy	0	0	0
Preoperative immunotherapy	0	5	5
Cardiac medical history	1	23	24
Respiratory medical history	1	11	12
Haematological medical history	0	4	4
History of diabetes mellitus	0	6	6
Pre-operative haemoglobin (mean; g/L)	126	129	
ASA Grade 3	0	1	1
ASA Grade 2	3	119	122
ASA Grade 1	0	1	1
Total	3	121	124

Group and save rates

Group and save (G&S) samples were sent to the majority of patients. Ninety-five patients (76.6%) had one sample sent before reconstruction, while 21 patients (16.9%) had two samples sent, and eight patients (6.5%) had no G&S samples sent preoperatively. Twenty patients (16.1%) had a sample sent on the day of the reconstructive operation. Four G&S samples (2.9%) were rejected before testing due to technical problems, including mislabelling of the sample bottle.

## Discussion

Background and transfusion cases

Preoperative blood testing is performed through a combination of protocol and historical practice. We aim to take an evidence-based approach to sampling to ensure efficient use of resources. Previously, Macdonald et al. reported on the transfusion rate in a similar group of patients [10]. No significant differences in patient or operative factors were identified between their study and our cohort. Of their 130 patients, 3.8% required red cell transfusion. Our rate, 2.4%, is consistent with this and other recent studies [10,11], and significantly lower than historical studies (19% [12] and 80% [13]). This apparent drop over time may reflect changing practices such as careful patient selection, improved operative techniques, or evolving transfusion protocols.

We routinely measure haemoglobin levels on day one postoperatively. In line with the Enhanced Recovery After Surgery (ERAS) protocol for breast reconstruction, at this point, the patient is mobilised, encouraged to resume oral intake, has the forced-air patient warmer removed, and the urinary catheter removed if it is safe to do so. Red cell transfusion is considered when postoperative haemoglobin is below 80 g/L, if the patient experiences profound clinical symptoms of anaemia such as dizziness, or shows signs such as haemodynamic instability or evidence of flap compromise due to anaemia. The decision is made by a senior registrar or consultant, given reports of a strong association between blood transfusion and failure of the microanastomosis in the context of free flaps [14].

In this study, we found a low transfusion rate, with only three out of 124 patients (2.4%) requiring transfusion. All those transfused had undergone DIEP-based reconstructions immediately following oncological resection. All received routine postoperative venous thromboembolism prophylaxis but no other anticoagulants or platelet inhibitors. None of these patients had preoperative G&S samples sent to the laboratory. Previous studies have identified bilateral reconstruction [11,12], longer operative duration [12], and preoperative anaemia [13] as factors increasing the risk of transfusion. We found no statistically significant differences in preoperative factors such as medical comorbidities, ASA grade, preoperative oncological treatment, or preoperative haemoglobin levels between those who received a transfusion and those who did not.

In the first case, the patient underwent bilateral removal of breast implants, capsulectomies, and unilateral DIEP-based reconstruction. On the first day postoperatively, a breast haematoma was diagnosed clinically, and the patient underwent exploration, evacuation of the haematoma, and haemostasis under general anaesthesia. No specific bleeding point was identified. On day two post-reconstruction, haemoglobin was noted to be 71 g/L, and two units of red blood cells were prescribed. The two G&S samples were sent at the time of the second operation; crossmatched red blood cells were requested and transfused 24 hours later. The patient was discharged and made a good postoperative recovery without further complications.

The second patient underwent left mastectomy, sentinel lymph node biopsy, and breast reconstruction with a DIEP flap. Around 60 hours postoperatively (day three), the patient noted increasing pain at the surgical site and returned to theatre for a washout of a breast haematoma of approximately 300 millilitres in volume. A small bleeding vessel from the lateral mastectomy skin flap was identified, along with other small bleeding sites, and haemostasis was achieved with electrosurgery. Two units of red blood cells were administered during this operation. The haemoglobin level was 129 g/L preoperatively, 94 g/L immediately before returning to theatre, and dropped to 70 g/L on arterial blood gas testing intraoperatively. The haemoglobin increased to 105 g/L immediately after the operation and transfusion, declining to 92 g/L two days later at the time of discharge, with no evidence of further bleeding.

The third patient underwent unilateral mastectomy for carcinoma and immediate DIEP-based reconstruction. The preoperative haemoglobin level was 107 g/L. On day one postoperatively, the haemoglobin level was 78 g/L, with no external evidence of bleeding. Two units of crossmatched red blood cells were administered later that evening and the next day. The haemoglobin increased to 104 g/L within 24 hours of the second unit being transfused.

G&S processing time

The presence of AARCAbs potentially increases the risk of transfusion reactions, usually resulting in a delayed haemolytic reaction. AARCAbs have a low prevalence of 1 to 2% [15-17]. The risk of developing AARCAbs arises after exposure to allogenic red blood cells, commonly through pregnancy, transfusion, or transplantation. Even in multiply transfused haematology patient populations, the group at highest risk for developing AARCAbs, the prevalence increases to only 12% [18]. Acute transfusion reactions occur rarely, in 0.5 to 3% of transfusions [19]. The risk of serious harm occurring through transfusion is described in the national 2023 Serious Hazards of Transfusion (SHOT) report as one per 11,000 blood components issued [20].

Our laboratory provided the local processing times for G&S samples and crossmatch processing [21]. For the initial G&S sample processing, the time required is 60 minutes; the requisite two separate samples (to confirm the correct patient) can be processed simultaneously. If no AARCAbs are identified, blood issue can be completed within 15 minutes using either electronic issue or immediate spin crossmatch. The process is significantly elongated if atypical antibodies are identified at any point. Antibody screens can take from 60 minutes to five days if referral to a reference laboratory is required. Blood issue can similarly take between 60 minutes and several days if atypical antibodies are identified.

In summary, for those without atypical antibodies, crossmatched red cell units can usually be made available on the same day. However, if a patient has atypical antibodies, testing and cross-matching can potentially take several days.

Financial cost

The cost of processing one G&S sample in our laboratory is £12.70. The cost of equipment is approximately £2. Labour and equipment costs are negligible when the sample is drawn simultaneously with other blood tests, such as routine preoperative tests. If sent alone, such as for a second G&S sample, the labour cost is approximately £3.40 (15 minutes of phlebotomist time, Band 3 £13.61/hour (2022/23 pay scale, according to local trust advert), not including employment, HR, or administrative costs) [22,23]. The total cost for all G&S testing in this cohort was therefore approximately £1853.

The cost of processing these samples must be weighed against the cost of delayed care if they are not sent. No delay in care was identified in our study. The cost per day of one hospital bed, excluding treatment costs, is £345 [24]; thus, even one extra day of admission per transfused patient is outweighed by the cost of G&S testing performed in this group.

Limitations of this study

This is a retrospective study and may therefore include some bias in recruitment and data collection. For example, there may have been loss to follow-up or missed patients during the screening process. We have aimed to minimise this by following a pre-determined study protocol, recruiting all patients over a limited one-year period, and maintaining broad inclusion criteria related to any breast reconstruction with a free flap. As all study data were stored on and collected from the electronic patient notes, the completeness of the data was high. We therefore expect the risk of these biases to be low. In addition, much of the data collected would have been documented and recorded at the point of care; therefore, some of the data fields can no longer be verified.

This study is restricted to our centre’s experience, located in London. As a result, our study results and conclusions may not be generalisable to diverse patient cohorts or operations. Furthermore, should operative factors or transfusion practices change, these results would likely also become unreliable.

Our sample size and duration of data collection were chosen to ensure minimal missing data and to reduce potential bias (the Trust's transfusion system was changed shortly before the beginning of our data collection period). This approach also aimed to minimise the effects of changing practices, such as restrictive transfusion protocols. However, this sample was not large enough to identify differences between subgroups. Further study of any subgroup differences is therefore recommended.

We have not assessed the effect of operative factors during mastectomy and reconstruction on blood transfusion rates or the volume of intraoperative blood loss. These factors could be an important focus for future research to reduce the use of valuable red blood cell units and to limit patient exposure to the morbidity associated with both volume loss and red blood cell transfusion. Addressing these factors would require a significantly larger sample size and considerable resources for data collection.

In addition, the change in protocol we recommend, namely, reducing the use of G&S sampling preoperatively, is subject to external validation outside this cohort of patients. We aim to conduct this study prospectively to monitor the effect of these changes.

## Conclusions

Patients undergoing breast reconstruction with a free flap in our centre have a low transfusion rate of 2.4%. None of these transfusions were emergent, nor did any require the major haemorrhage protocol. None had G&S sampling before their reconstructive operation.

To maximise efficiency in resource use, we recommend that no G&S samples be routinely sent for patients undergoing either immediate or delayed breast reconstruction with a free flap. For the subgroup with risk factors for atypical anti-red cell antibodies, one G&S sample should be sent preoperatively.
